# Association of cumulative early medical factors with autism and autistic symptoms in a population-based twin sample

**DOI:** 10.1038/s41398-022-01833-0

**Published:** 2022-02-22

**Authors:** Torkel Carlsson, Mina Rosenqvist, Agnieszka Butwicka, Henrik Larsson, Sebastian Lundström, Pei-Yin Pan, Karl Lundin Remnélius, Mark J. Taylor, Sven Bölte

**Affiliations:** 1 Center of Neurodevelopmental Disorders (KIND), Centre for Psychiatry Research; Department of Women’s and Children’s Health, Karolinska Institutet & Stockholm Health Care Services, Region Stockholm, Stockholm, Sweden; 2 Child and Adolescent Psychiatry, Stockholm Health Care Services, Region Stockholm, Stockholm, Sweden; 3 PRIMA Child and Adult Psychiatry, Stockholm, Sweden; 4 Department of Medical Epidemiology & Biostatistics, Karolinska Institutet, Stockholm, Sweden; 5 Child and Adolescent Psychiatry Stockholm, Stockholm Health Care Services, Region Stockholm, Stockholm, Sweden; 6 Department of Child Psychiatry, Medical University of Warsaw, Warsaw, Poland; 7 Schools of medical sciences, Örebro University, Örebro, Sweden; 8 Gillberg Neuropsychiatry Centre, University of Gothenburg, Gothenburg, Sweden; 9 Centre for Ethics, Law, and Mental Health, University of Gothenburg, Gothenburg, Sweden; 10 Curtin Autism Research Group, Curtin School of Allied Health, Curtin University, Perth, Western Australia

**Keywords:** Pathogenesis, Physiology

## Abstract

Although highly heritable, environment also contributes to the etiology of autism spectrum disorder (ASD), with several specific environmental factors previously suggested. A registry-linked population-based twin cohort of 15,701 pairs (586 individuals with an ASD diagnosis), was established within the Child and Adolescent Twin Study in Sweden. Participants were evaluated for autistic symptoms at age 9 using the Autism-Tics, ADHD and other Comorbidities parental interview. A series of binary cut-offs indicated whether participants scored over various ASD symptom percentiles. Three early medical factors previously associated with ASD, beyond familial confounding (low birth weight, congenital malformations and perinatal hypoxia), were summed up creating an individual cumulative exposure load. A series of unconditional logistic regressions between all individuals and conditional regressions within twin pairs were performed for each outcome and exposure level. Between all individuals increasing cumulative early exposure loads were associated with increasing risk of ASD diagnosis (OR 3.33 (95%CI 1.79–6.20) for three exposures) and autistic symptoms (ranging from OR 2.12 (1.57–2.86) for three exposures at the 55th symptom percentile cut-off to OR 3.39 (2.2–5.24) at the 95th). Within twin pairs, the association between three exposures and an ASD diagnosis remained similar, but not statistically significant (OR 2.39 (0.62–9.24)). Having a higher load of early cumulative exposure was consistently associated with autistic symptoms after adjusting for familial confounding and sex (OR 3.45 (1.66–7.15) to OR 7.36 (1.99–27.18)). This study gives support to the cumulative stress hypothesis of ASD, and the dimensional model regarding environmental exposures, after adjustment for familial confounding.

## Introduction

Autism spectrum disorder (ASD) is a neurodevelopmental disorder characterized by atypical social communication and interaction along with restricted, repetitive behaviors and sensory alterations that affect adaptive functioning in major life areas [1]. There is growing consensus today that ASD forms the extreme end of a continuum of autistic traits in the general population, with the clinical and nonclinical phenotypes having overlapping, genetically driven neurodevelopmental origins [2]. The heritability of clinical ASD is estimated at 83–95% [3–5] and 61–78% for autistic traits [2], leaving space for environmental contributions to ASD etiologies. These studies suggest that environmental factors are largely those that contribute to differences between twins.

Several environmental factors for ASD have been suggested, such as advanced maternal and paternal age, and the pregnancy related factors of enhanced steroidogenic activity, immune activation, maternal valproate intake, maternal treatment with selective serotonin reuptake inhibitors and maternal diabetes, toxic chemical exposure, and possibly altered zinc–copper cycles [6]. Different models of underlying genetic and environmental mechanisms may thus apply to ASD. Regarding the environment, the cumulative stress hypothesis proposes that vulnerability for a given condition, such as ASD [7], is enhanced if adversities accumulate during early life [8]. Within the three-hit concept [9], another generic etiological model, cumulative early life stress is considered a second hit, subsequent to genetic predisposition (first hit) and followed by later-life environment (third hit), with evidence for this model applying in ASD so far found in animal studies [10–12]. According to the dimensional model of ASD [13], these underlying genetic and environmental factors are assumed to form a continuous distribution of liability to a categorical outcome, such as a diagnosis of ASD [14]. To the best of our knowledge a cumulative environmental effect on ASD has only been studied twice before. In a smaller genetically informed rigorously phenotyped twin sample, we have previously found that cumulative exposure to early medical factors is associated with both a diagnosis of ASD, and with autistic symptoms [15] and a recent study found that cumulative exposure to phtalate mixtures in pregnancy is associated with autistic traits [16], thus supporting this hypothesis.

One important bias in the study of the association between environmental exposures and ASD is familial confounding. Familial confounding refers to both unmeasured genetic and environmental factors, shared within families and which make family members similar. Since many exposures are in themselves under genetic influence, such as paternal age and the use of antidepressive medication during pregnancy, it cannot be ruled out that an observed association is confounded by familial links between the exposure and the outcome, rather than it being causal. For example, the association between ASD and antidepressive medication during pregnancy is largely attenuated when accounting for shared genetic and environmental factors [17–19], but paternal age, with a heritability of 33% [20], is still associated with ASD after accounting for familial confounding [21]. Twin studies can help to investigate whether an observed association is due to familial confounding [22], because genetic and environmental factors shared within twins will be adjusted for. In a recent systematic review of twin and sibling studies only low birth weight, congenital malformations, perinatal hypoxia, and advanced paternal age, were found to be associated with ASD beyond familial confounding [23].

To date, no sufficiently large and genetically informative study has addressed familial confounding in relation to cumulative environmental exposure and ASD. Therefore, the objective of this study was to test the hypothesis of a cumulative effect of environmental exposures on ASD and autistic symptoms using a large population-based twin cohort, while controlling for familial confounding. We sought to examine the effects of the early medical factors of low birth weight, congenital malformations and perinatal hypoxia in ASD and autistic symptoms identified in the recent systematic review [23].

## Subjects and methods

### Participants

This study and the linkage of samples with registries was approved by the Regional Ethical Review Board in Stockholm. Twins were recruited from the longitudinal, population-based Child and Adolescent Twin Study in Sweden (CATSS), which was initiated in 2004 [24]. In CATSS, all parents of twins aged 9 years (earlier cohorts included 12-year-olds) that are born in Sweden are invited to report on the twins’ neurodevelopmental symptoms using a validated structured interview. This study included a cohort of 15,701 twin pairs, with data collected from individuals born in every year between 1992 and 2008 (Table 1). CATSS has an answering frequency of 75% since 2004, and selected sample characteristics have been shown to be representative for the general population in Sweden [2]. Zygosity was determined by DNA analysis. For twins without DNA samples an algorithm based on five questions on twin similarity was used. Twins were only assigned zygosity through the algorithm method if the test achieved a 95% probability of producing a correct categorization [24]. CATSS participants’ consent was inferred by them completing the interviews.

**Table 1:** Sample characteristics.

Percentile		Whole sample	No exposure	Exposure level = 1	Exposure level = 2	Exposure level = 3
Mean A-TAC score (SD)		0.85 (1.65)	0.79 (1.57)	0.98 (1.83)	1.14 (2.04)	1.63 (2.43)
Total *N* individuals (%)		31,402 (100.0)	24238 (77.2)	5101 (16.2)	1872 (6.0)	191 (0.6)
*N* with ASD diagnosis (%)		586 (1.9)	412 (1.7)	104 (2.0)	59 (3.2)	11 (5.8)
*N* of individuals at each percentile of A-TAC (%)	95th	1565 (5.0)	1071 (4.4)	328 (6.5)	141 (7.6)	25 (13.3)
	90th	3130 (10.0)	2206 (9.1)	615 (12.1)	272 (14.6)	37 (19.7)
	85th	4695 (15.0)	3392 (14.0)	863 (17.0)	385 (20.6)	55 (29.3)
	80th	6260 (20.0)	4585 (19.0)	1124 (22.1)	480 (25.7)	71 (37.8)
	75th	7825 (25.0)	5774 (23.9)	1388 (27.3)	583 (31.2)	80 (42.6)
	70th	9390 (30.0)	6962 (28.8)	1658 (32.6)	681 (36.5)	89 (47.3)
	65th	10956 (35.0)	8167 (33.8)	1918 (37.7)	775 (41.5)	96 (51.1)
	60th	12522 (40.0)	9355 (38.7)	2183 (42.9)	878 (47.1)	106 (56.4)
	55th	14088 (45.0)	10,534 (43.6)	2461 (48.4)	977 (52.4)	116 (61.7)
Median birthyear (IQR)		2000 (1996, 2004)	2000 (1996, 2004)	2000 (1996, 2004)	2001 (1996, 2004)	1998 (1994, 2004)
*N* of females (%)		15473 (49.3)	12,167 (50.2)	2345 (46.0)	874 (46.7)	87 (45.5)
*N* of MZ twins (%)		9446 (30.1)	6943 (28.6)	1681 (33.0)	742 (39.6)	80 (41.9)
*N* of DZ twins (%)		21,956 (69.9)	17,295 (71.4)	3420 (67.0)	1130 (60.4)	111 (58.1)

*A-TAC* the Autism–Tics, ADHD and Other Comorbidities Inventory, *MZ* monozygotic, *DZ* dizygotic

### Exposures

**Fig. 1:**
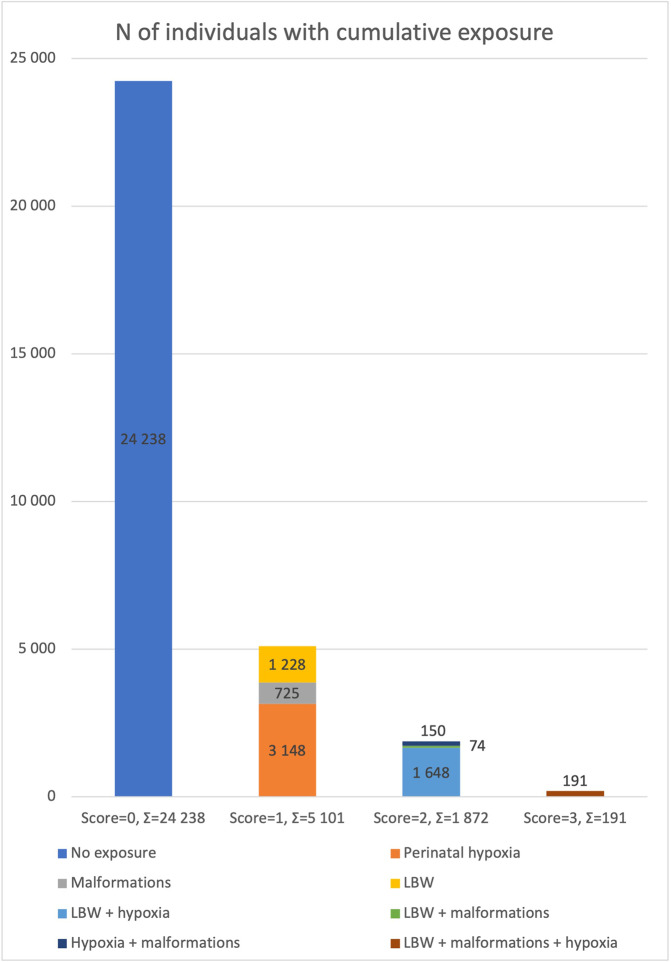
Bar chart of the sample distribution of cumulative exposure load of early medical factors. *LBW* gestational age adjusted low birth weight.

Low birth weight, congenital malformations and perinatal hypoxia were examined in this study. These early adverse environmental factors were chosen as they have previously yielded associations with ASD beyond familial confounding [23]. Since paternal age does not differ within twin pairs this factor was not included. We obtained detailed obstetric and neonatal information, as well as all diagnosis codes of interest for all participants throughout their lives, by linking CATSS to the Swedish Medical Birth Register (MBR), which covers more than 90% of all deliveries in Sweden [25], and the National Patient Register (NPR), which records inpatient diagnoses (with nationwide coverage from 1987) and outpatient diagnoses from 2001 [26], with follow-up to November 30, 2018. We created a binary variable to indicate whether each factor was present or not for each participant by identifying diagnosis codes for each medical adversity according to the International Classification of Diseases, Ninth (ICD-9; 1987–1996) and Tenth Revision (ICD-10;1997–2013), and from relevant obstetric information from the MBR and CATSS parental interview using SAS version 15.1 (see Supplementary Table 1 for complete list of codes for each factor). We regressed birth weight on gestational age, creating a binary gestational age adjusted low birth weight variable in our population-based sample, assigning the value “1” to the lowest 10th percentile. To create an ordinal cumulative exposure load variable of early medical factors the presence of binary factors was summed up for each participant. The sample distribution of the cumulative exposure ranged from 24,238 participants with a 0 score, to 191 with a score of 3 (Table 1, Fig. 1).

### Outcomes

#### Diagnosis of ASD

All diagnosis codes for pervasive developmental disorders under ICD-10 code F84 were extracted from the NPR and coded binary for each participant, excluding Rett Syndrome (F84.2), other childhood disintegrative disorders (F84.3), and overactive disorder associated with intellectual disability and stereotyped movements (F84.4). The validity of the registry-based diagnosis is high [27, 28].

#### Autistic symptoms

In CATSS, all participants are evaluated for autistic symptoms at the age of 9 or 12 using the Autism-Tics, ADHD and other Comorbidities inventory (A-TAC) [29]. Its validity and predictive properties are well established through clinical and population-based samples [30, 31]. Seventeen items address ASD and can be answered yes (scored as 1), yes, to a certain degree (0.5), or no (0). The sample distribution of the score is skewed, ranging from 0 to 17, with a 0 score for the lower 55th percentile of the sample. To generate binary ASD symptom level variables from the A-TAC score, nine cut-offs were created for every 5th percentile, from the 55th to the 95th, indicating whether participants scored over the percentile cut-off for autistic symptoms or not. Descriptive statistics for each percentile cut-off are shown in Table 1.

### Statistics

A series of logistic regressions were performed with each ASD symptom level outcome, and ASD diagnosis outcome respectively, regressed on each level of cumulative exposure load of early medical factors using generalized estimation equations (GEE) with robust sandwich estimators to account for related individuals in the sample, from the *R* package drgee [32] and *R* version 4.0.4. As a first step, unadjusted between-individual regressions were performed, followed by between-individual regressions adjusting for sex and birth year. Next, conditional logistic regressions were performed, estimating the within-pair effect. This adjusts for all factors shared within twins; in monozygotic (MZ) twins this includes all genetic and shared environmental influences, while in dizygotic (DZ) twins it includes approximately 50% of genetic influences and all shared environmental influences. As such, these models adjust for familial confounding. Finally, conditional logistic regressions within twins were performed adjusting for sex [33]. To evaluate the occurrence of statistically significant exposure level differences regarding dose response associations, all regressions were repeated comparing each exposure level respectively. All analyses were also performed split by sex (*n* = 10,254 twin pairs), with opposite sex twin pairs excluded, since no within twin pair analysis can be performed on twins split into different sex groups. As previously discussed [33], the informative twin pairs in conditional logistic regressions are those simultaneously discordant for exposure/covariates and outcome, see Supplementary Tables 2 and 3. Regarding an ASD diagnosis, 54 twin pairs were positively discordant for both outcome and exposure, that is the twin with ASD-diagnosis having more exposure than their co-twin without a diagnosis, and 58 twin pairs were discordant in the reversed direction, that is the twin with the ASD diagnosis having less exposure than their unaffected co-twin. Regarding the autistic symptom cut-offs there were 164 twin pairs positively discordant for both outcome and exposure at the 95th percentile, steadily rising to 578 twin pairs at the 55th percentile, with a consistently lower number of reversed discordances at every symptom cut-off.

### Sensitivity analyses

To rule out the possibility of one single factor explaining the association all analyses were repeated with each factor respectively excluded. All regressions were also performed for each factor separately. Also, since twin-to-twin transfusion syndrome (TTTS) carries a risk for altered neurodevelopment [34], all regressions were repeated with *n* = 93 twin pairs excluded, were either one or both twins in the pair had received a diagnosis of TTTS.

## Results

### Between all participants

**Fig. 2:**
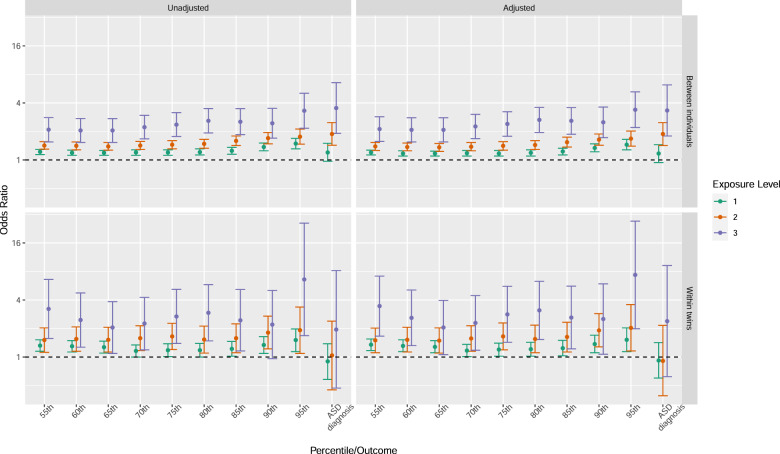
Between individual (upper panels) and within twin-pair (lower panels) associations between the cumulative exposure load of early medical factors and a diagnosis of ASD, and being above each percentile cut-off of autistic symptoms, respectively. Forest plots illustrating odds ratios (ORs, dots) and 95% confidence interval (CI, bars) for unadjusted associations to each exposure level (left panels), and sex and birth year adjusted between individual (upper right panel) and familial confounding and sex adjusted (lower right panel) within twin associations.

A higher load of early cumulative exposure was associated with a diagnosis of ASD, with a sex and birth year adjusted odds ratio [OR] of 1.17 (95%CI, 0.94–1.45) for one exposure, OR 1.88 (1.42–2.48) for two exposures and OR 3.33 (1.79–6.20) for three exposures. Furthermore, a higher load of early cumulative exposure was consistently associated with having more autistic symptoms, ranging from OR 1.20 (1.13–1.27) at the 55th autistic symptom percentile to OR 1.45 (1.28–1.65) at 95th percentile, for one exposure, from OR 1.39 (1.26–1.53) to OR 1.68 (1.40–2.02), for two exposures, and from OR 2.12 (1.57–2.86) to OR 3.39 (2.2–5.24) for three exposures (Fig. 2, Supplementary Table 4). Regarding dose response associations, statistically significant differences between exposure levels were observed between all levels.

### Within twin pairs

The association between one and two exposures and a diagnosis of ASD seen in the unconditional logistic regression attenuated towards null, with odds ratios just below 1, when adjusted for familial confounding and sex, with OR 0.92 (0.60–1.42) for one exposure, and OR 0.91 (0.39–2.16) and for two exposures (Fig. 2, Supplementary Table 4). For three exposures, however, the odds ratio remained similar to that of the unconditional association, OR 2.39 (0.62–9.24), but was not statistically significant. No exposure level difference was statistically significant.

Higher loads of early cumulative exposure in one twin was consistently associated with having more autistic symptoms than their co-twin at every symptom cut-off, after adjusting for familial confounding and sex, with increasing ORs with each increasing symptom level ranging from 1.35 (1.17–1.55) at the 55th symptom percentile to OR 1.52 (1.14–2.03) at 95th percentile for one exposure, from OR 1.50 (1.11–2.02) to OR 2.03 (1.16–3.58) for two exposures and from OR 3.45 (1.66–7.15) to OR 7.36 (1.99–27.18) for three exposures (Fig. 2, Supplementary Table 4). Statistically significant exposure level differences were only observed for level 1 to 3 in six of the ten autistic symptom severity cut-offs.

**Fig. 3:**
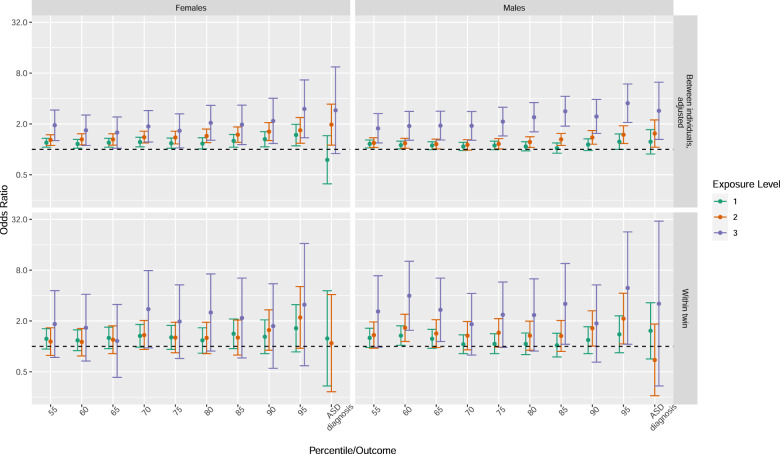
Birthyear adjusted between individual (upper panels) and familial confounding adjusted within twin-pair (lower panels) associations between the cumulative exposure load of early medical factors and a diagnosis of ASD, and being above each percentile cut-off of autistic symptoms, respectively. Forest plots illustrating odds ratios (ORs, dots) and 95% confidence interval (CI, bars) from conditional regressions on females (left panels) and males (right panels), *n* = 10,254 twin pairs, excluding opposite sex twins.

The result split by sex was in line with the whole sample, with similar results for males and females, although with wider 95% confidence intervals for females, which overlapped with 1 (Fig. 3, Supplementary Table 5).

### Sensitivity analysis

Similar results were seen with each factor removed respectively (Supplementary Table 6, Supplementary Fig. 1), ruling out one factor explaining all of the associations between cumulative exposure and ASD, although malformations was the single factor driving the associations the most (Supplementary Table 7, Supplementary Fig. 2). Excluding twin pairs with a diagnosis of twin-to-twin transfusion syndrome did not affect the results, indicating that this twin related syndrome did not explain the result (Supplementary Table 8, Supplementary Fig. 3).

## Discussion

This study aimed to test the cumulative stress hypothesis of ASD and assessed whether the association between cumulative exposure to early medical factors and ASD and symptoms of ASD, is due to familial confounding. By summing up the cumulative load of strictly selected early environmental factors for ASD we saw increasing associations with a clinical ASD diagnosis for each added exposure. These associations attenuated after adjustment for familial confounding for the lower levels of cumulative exposure but remained similar after adjustment for familial confounding for the highest level of exposure, although not statistically significant. However, this might have been because of low power. Regarding later autistic symptom severity at nine years of age, the associations persisted after adjustment for familial confounding. Therefore, this study, in line with the recent findings by Day et al. [16], gives support to the cumulative stress hypothesis of ASD, although with the notion that familial confounding might explain the association between cumulative exposure to early risk factors and clinical ASD, but not level of ASD symptoms.

The consistency of our findings over a series of autistic symptom severity cut-offs gives further support to the dimensional nature of ASD [35]. Twin studies show that there is genetic overlap between a diagnosis of ASD and autistic symptoms [3, 36], and molecular genetic studies show common genetic variant overlap [37]. However, the dimensional model could also include environmental influences. To date, little is known about whether environmental overlap exists across differing symptom severity levels and a diagnosis of ASD, although environmental overlap has been shown to be possible [3, 36]. To the best of our knowledge, this study is the first to demonstrate that there might be continuity in environmental influences across ASD symptom severity. However, an important caveat that needs to be addressed is the different pattern of findings for clinical ASD, especially with regards to a lower level of cumulative exposure. This opens the possibility that the ASD diagnosis could be discontinuous in relation to the environmental exposures we have studied, but since our cumulative load of early medical factors were summed up by only three exposures, a replication of the results with different exposures, is needed.

The presence of an association between the cumulative load of early medical factors with later autistic symptom severity beyond familial confounding suggests that cumulative exposure to early environmental risks might be on the etiological pathway to ASD. Within the three-hit concept, the cumulative load of environmental contributions is designated as a second hit, that together with a first hit of genetic predisposition, forms gene-environment interactions during critical phases of perinatal and juvenile brain development [9]. Little is known about gene-environment interactions in ASD [38, 39]. Specific twin models where genetic influences on the phenotypic variance are expressed as a linear function of the environment [40], have been applied before to explore gene-environment interactions in phenotypes other than ASD [41, 42]. To date, clear evidence that gene-environment interactions contribute to ASD etiology is absent [43], although shown to be important in animal studies [44, 45]. To further understand the role of the cumulative load of early environmental risk factors and ASD, gene-environment interactions need to be addressed in future research.

The attenuation seen after adjustment for familial confounding regarding associations to clinical ASD, especially the associations to the lower levels of cumulative exposure, is a clinically intriguing finding. Even though low power could explain some of this attenuation, familial factors may contribute to the associations found between all individuals, meaning children of parents with autism may be more likely to experience the accumulation of these early medical exposures, thereby pointing to a need for clinical awareness as well as more research on the effects of accumulating environmental exposure.

For our hypothesis driven approach, we only choose environmental factors that in a recent comprehensive systematic review had been found to be associated with ASD beyond familial confounding [23]. Since these environmental factors were early medical factors, we were able to measure them by linking national medical registries to our sample. This might be an example of the streetlight effect [46]—our list of factors stemming from prior research investigating only what has been possible to investigate. Even though this study is still selective and may not have included other pertinent environmental exposures, it shows the importance of the accumulation of environmental factors beyond familial confounding.

As previously noted, evidence for the three hit concept in ASD etiology has only been found in animal studies [11, 12]. To further expand our result of a cumulative effect to a wider range of environmental factors, human-induced pluripotent stem cells (hiPSCs) and brain organoids are potential tools where human studies would be unethical or unpractical to conduct [47]. To date, no study has been conducted on environmental impacts on hiPSCs or brain organoids derived from subjects with ASD, but a few studies in recent years have pointed out the potential of studying environmental factors’ impact on neurodevelopment using hiPSCs [48–50]. With the future development of reliable ASD hiPSCs and brain organoids, the effect of environmental factors could be possible to test experimentally. Then, suggested underlying mechanisms within the cumulative stress hypothesis involving for example environmental pollutants [51], endocrine disruptors [38], or stress hormones [9] would be possible to study within the framework of the three-hit concept of genetic vulnerability interplay with early and pervasive factors in the environment. Our study emphasizes the importance of studying the cumulative effects of such environmental factors.

### Strengths and limitations

Our study has several strengths. First, to be conservative, we chose potential environmental risk factors that had been previously shown to be associated with ASD after adjustment for familial confounding. This put us in a unique position to interpret *any* association, even a weak one, as a potential new piece of evidence. Nevertheless, since the associations found are weak, we stress the importance of interpreting our findings cautiously, particularly in face of the high heritability of ASD. Second, our large population-based twin sample enables the investigation of even weak, yet important, environmental contributions beyond familial confounding. Third, as the sensitivity analyses show, the importance of environmental risk accumulation is consistent also after excluding each single exposure. Forth, by measuring both diagnosis and symptoms of ASD we were able to include a categorical as well as a dimensional model of ASD in our study.

Limitations need to be addressed. First, the cumulative score was created by summing exposures based on them being present or absent, and therefore, we were not able to account for the possibility of different effect sizes for each exposure. Second, as for all observational studies the risk of residual bias prohibits far reaching causal interpretations. Specifically, we cannot completely rule out confounding by child specific genetic effects. By comparing twins we completely rule out the effect of parental genetics, but the exposures studied might be influences by child genetics as well. The only way to rule out confounding from child genetics is to compare the within pair difference of MZ twins. While MZ twins are a part of our sample, we unfortunately did not have the power to look at MZ and DZ twin separately. Third, generalizability from twins to singletons is important to consider, especially since we saw a slightly higher percentage of monozygotic pairs in the higher exposure load groups (Table 1). However, the literature on the role of zygosity for perinatal outcomes is scant. Zygosity has in a previous retrospective study been linked to lower birth weight and more prematurity [52], while in a later study the effect of zygosity was less clear [53]. It is also worth highlighting that the results did not differ when twin pairs with twin-to-twin transfusion syndrome were excluded. Fourth, for the adjusted within pair analyses the informative twin pairs are primarily those that are simultaneously discordant for outcome and exposure, together with those simultaneously discordant for outcome and covariates [33]. Even with our large sample size, the number of exposure and outcome discordant pairs were quite low, especially with regards to a diagnosis of ASD as the outcome, and furthermore when considering the number of pairs discordant in the reversed direction. Finally, as a source of potential residual bias, measurement error at the within-pair level needs to be kept in mind. However, it is well established that birth weight is a risk factor that can be studied within twin pairs [15, 54–56], perinatal hypoxia has previously been studied both in a twin setting [57], and in sibling settings [58–60] and malformations as a risk factor has been studied within sibling pairs [61–63]. Furthermore, the presence of a measurement error or misclassification of a binary variable leads to an attenuation of both the between individuals and the within pair association, compared to the true association, with within-pair associations tending to be more attenuated than between-pair associations [64].

## Conclusion

This study gives support to the cumulative stress hypothesis of autism spectrum disorder beyond familial confounding. It demonstrates that there might be continuity in environmental influences across ASD symptom severity giving support to the dimensional model of ASD. In line with the three hit concept, we suggest future research to focus on gene-environment interaction using both observational twin studies and experimental human-induced pluripotent stem cells, to further explore the cumulative nature of early environmental stress in the etiology of ASD.

## Supplementary information

Supplementary information

## Data Availability

Custom written SAS and *R* scripts used for statistical analyses can be provided upon request.
