# Supplementary material for: Association of cumulative early medical factors with autism and autistic symptoms in a population-based twin sample
Source: Transl Psychiatry. 2022 Feb 22;12:73. doi: 10.1038/s41398-022-01833-0 (PMC8863884; doi:10.1038/s41398-022-01833-0)
Supplement: Supplementary file 1 — Supplementary information [file 41398_2022_1833_MOESM1_ESM.doc]

Supplementary information

Table of content

[Supplementary Table 1. Complete list of diagnosis codes for each exposure factor 3](#__RefHeading___Toc94196459)

[Supplementary Table 2. Sample distribution of exposure discordant twin pairs 5](#__RefHeading___Toc94196460)

[Supplementary Table 3. Sample distribution of doubly discordant twin pairs for both exposure and outcome 6](#__RefHeading___Toc94196461)

[Supplementary Table 4. Between individual and within twin pair associations of the cumulative load of early medical adversities and ASD and autistic symptom cut-offs 7](#__RefHeading___Toc94196462)

[Supplementary Table 5. Split by sex – Associations between the cumulative exposure load of early medical factors and a diagnosis of ASD, and being above each percentile cut-off of autistic symptoms, respectively. N*=* 10,254 twin pairs, excluding opposite sex twins. 8](#__RefHeading___Toc94196463)

[Supplementary Table 6. Birthyear adjusted between individual and familial confounding adjusted within twin-pair associations between the cumulative exposure load of early medical factors and a diagnosis of ASD, and being above each percentile cut-off of autistic symptoms, with each factor removed respectively. 9](#__RefHeading___Toc94196464)

[Supplementary Figure 1. Cumulative exposure with each factor removed respectively 10](#__RefHeading___Toc94196465)

[Supplementary Table 7. Birthyear adjusted between individual and familial confounding adjusted within twin-pair associations for each exposure respectively and a diagnosis of ASD, and being above each percentile cut-off of autistic symptoms 11](#__RefHeading___Toc94196466)

[Supplementary Figure 2. Odds Ratios for each exposure respectively 12](#__RefHeading___Toc94196467)

[Supplementary Table 8. Between individual and within twin pair associations between the cumulative load of early medical factors and ASD and autistic symptom cut-offs. *n=93* twin pairs excluded, were either one or both twins in the pair had received a diagnosis of twin-to-twin transfusion syndrome. 13](#__RefHeading___Toc94196468)

[Supplementary Figure 3. Cumulative exposure excluding twins with twin-to-twin transfusion syndrome 14](#__RefHeading___Toc94196469)

# Supplementary Table 1. Complete list of diagnosis codes for each exposure factor

| **Exposure variable** | **ICD-10 codes** | **ICD-9 codes** | **Description** | **MBR^1^ information** | **CATSS-Question^2^** |
| --- | --- | --- | --- | --- | --- |
| Birth weight |  |  |  | Birth weight | What birthweight? |
|  |  |  |  | Gestational age |  |
| Malformations included codes | Q00-Q07 | 740-742 | Congenital malformations of the nervous system |  |  |
|  | Q10-Q18 | 743-744 | Congenital malformations of eye, ear, face and neck |  |  |
|  | Q20-Q28 | 745-747 | Congenital malformations of the circulatory system |  |  |
|  | Q30-Q34 | 748- | Congenital malformations of the respiratory system |  |  |
|  | Q35-Q37 | 749-750 | Cleft lip and cleft palate |  |  |
|  | Q38-Q45 | 751- | Other congenital malformations of the digestive system |  |  |
|  | Q50-Q56 | 752- | Congenital malformations of genital organs |  |  |
|  | Q60-Q64 | 753- | Congenital malformations of the urinary system |  |  |
|  | Q65-Q79 | 754-757 | Congenital malformations and deformations of the musculoskeletal system |  |  |
|  | Q80-Q89 | 759- | Other congenital malformations |  |  |
| Minor malformations excluded codes | Q0461, Q0782 |  | Nervous system |  |  |
|  | Q101-Q103, Q105, Q135 | 74365 | Eye |  |  |
|  | Q170-Q175, Q179, Q180- Q182, Q184- Q187, Q1880, Q189 | 74411, 74412, 7443, 74491 | Ear, face and neck |  |  |
|  | Q2111, Q250 if GA <37 weeks, Q2541, Q256 if GA<37 weeks, Q261 | Q250, 7470 if GA <37 weeks | Congenital Heart Defects |  |  |
|  | Q309, Q320, Q331 | 74819 | Respiratory |  |  |
|  | Q381, Q382, Q3850, Q400, Q401, Q4021, Q430, Q4320, Q4381, Q4382 | 7500, 7506 | Digestive system |  |  |
|  | Q610, Q627, Q633 |  | Urinary |  |  |
|  | Q523, Q525, Q527, Q53, Q540, Q5520, Q5521 | 7525, 75260 | Genital |  |  |
|  | Q653-Q656, Q662-Q669, Q670-Q678, Q680, Q6810, Q6821, Q683- Q685, Q7400 | 75432, 75452, 75460, 75473, 75481, 75560 | Limb |  |  |
|  | Q825, Q8280, Q8281 | 75731, 75738 | Congenital skin disorders |  |  |
| Perinatal hypoxia included codes | P20 | 7680-7684 | Intrauterine hypoxia |  | Breathing problems as newborn? |
|  | P21 | 7685, -86, -89, 7708 | Birth asphyxia |  |  |
|  | P22 | 769, 7706, -08 | Respiratory distress of newborn |  |  |
|  | DG001, DG007 | V9127 | CPAP/BPAP |  |  |
|  | DG002, DG021 | 9201 | Respirator |  |  |
|  | DG010 | V9128 | Manual ventilation |  |  |
|  | DG015 | V9125 | Oxygen treatment, spontaneous breathing |  |  |

^1^ The Medical Birth Register

^2^ Questions from the CATSS database parental interview

# Supplementary Table 2. Sample distribution of exposure discordant twin pairs

|  |  |  | **Exposure discordant** | | ***Each level of exposure discordancy*** | | | | | |
| --- | --- | --- | --- | --- | --- | --- | --- | --- | --- | --- |
|  | **Percentile** | **Whole sample** | **No** | **Yes** | ***0 to 1*** | ***0 to 2*** | ***0 to 3*** | ***1 to 2*** | ***1 to 3*** | ***2 to 3*** |
| **Total *N* of pairs** |  | 15 701 | 12 540 | 3161 | *2405* | *152* | *5* | *461* | *29* | *109* |
| ***N* of ASD discordant pairs (%)** |  | 452 (2.9) | 340 (2.7) | 112 (3.5) | *78 (3.2)* | *6 (3.9)* | *0 (0.0)* | *19 (4.1)* | *>5 (13.8)* | *5 (4.6)* |
| **N of discordant pairs at each percentile of A-TAC (%)** | 95th | 1138 (7.3) | 867 (6.9) | 271 (8.6) | *201 (8.4)* | *14 (9.2)* | *0 (0.0)* | *42 (9.2)* | *>5 (14.3)* | *10 (9.3)* |
|  | 90th | 2097 (13.4) | 1594 (12.8) | 503 (16.0) | *362 (15.1)* | *28 (18.4)* | *>5 (20.0)* | *85 (18.6)* | *7 (25.0)* | *20 (18.7)* |
|  | 85th | 2769 (17.7) | 2131 (17.1) | 638 (20.3) | *459 (19.2)* | *37 (24.3)* | *>5 (20.0)* | *107 (23.4)* | *11 (39.3)* | *23 (21.5)* |
|  | 80th | 3440 (22.0) | 2689 (21.5) | 751 (23.9) | *547 (22.8)* | *39 (25.7)* | *>5 (40.0)* | *122 (26.6)* | *10 (35.7)* | *31 (29.0)* |
|  | 75th | 3831 (24.5) | 2994 (24.0) | 837 (26.6) | *618 (25.8)* | *45 (29.6)* | *>5 (60.0)* | *129 (28.2)* | *11 (39.3)* | *31 (29.0)* |
|  | 70th | 4261 (27.3) | 3336 (26.7) | 925 (29.4) | *688 (28.7)* | *50 (32.9)* | *>5 (40.0)* | *141 (30.8)* | *10 (35.7)* | *34 (31.8)* |
|  | 65th | 4478 (28.7) | 3511 (28.1) | 967 (30.8) | *729 (30.5)* | *49 (32.2)* | *>5 (40.0)* | *143 (31.2)* | *10 (35.7)* | *34 (31.8)* |
|  | 60th | 4648 (29.7) | 3654 (29.3) | 994 (31.6) | *753 (31.5)* | *52 (34.2)* | *>5 (40.0)* | *146 (31.9)* | *10 (35.7)* | *31 (29.0)* |
|  | 55th | 4811 (30.8) | 3799 (30.4) | 1012 (32.2) | *772 (32.2)* | *47 (30.9)* | *>5 (40.0)* | *154 (33.6)* | *10 (35.7)* | *27 (25.2)* |

*ASD* autism spectrum disorder, *A-TAC* Autism–Tics, ADHD and Other Comorbidities Inventory

# Supplementary Table 3. Sample distribution of doubly discordant twin pairs for both exposure and outcome

|  | **Percentile** | **Doubly discordant** | | ***Each level of Exposure discordancy*** | | | | | |
| --- | --- | --- | --- | --- | --- | --- | --- | --- | --- |
|  |  | Type |  | *0 to 1* | *0 to 2* | *0 to 3* | *1 to 2* | *1 to 3* | *2 to 3* |
| ***N* of ASD discordant pairs (%)** |  | **Yes** | **54 (1.7)** | ***36 (1.5)*** | ***>5 (2.6)*** | ***0 (0.0)*** | ***8 (1.7)*** | ***>5 (13.8)*** | ***>5 (1.8)*** |
|  |  | No | 3049 (96.5) | *2327 (96.8)* | *146 (96.1)* | *5 (100.0)* | *442 (95.9)* | *25 (86.2)* | *104 (95.4)* |
|  |  | Reversed | 58 (1.8) | *42 (1.7)* | *>5 (1.3)* | *0 (0.0)* | *11 (2.4)* | *0 (0.0)* | *>5 (2.8)* |
| **N of discordant pairs at each percentile of A-TAC (%)** | 95th | **Yes** | **164 (5.2)** | ***120 (5.0)*** | ***10 (6.6)*** | ***0 (0.0)*** | ***23 (5.0)*** | ***>5 (10.3)*** | ***8 (7.3)*** |
|  |  | No | 2890 (91.4) | *2204 (91.6)* | *138 (90.8)* | *5 (100.0)* | *419 (90.9)* | *25 (86.2)* | *99 (90.8)* |
|  |  | Reversed | 107 (3.4) | *81 (3.4)* | *>5 (2.6)* | *0 (0.0)* | *19 (4.1)* | *>5 (3.4)* | *>5 (1.8)* |
|  | 90th | **Yes** | **290 (9.2)** | ***206 (8.6)*** | ***20 (13.2)*** | ***0 (0.0)*** | ***48 (10.4)*** | ***>5 (13.8)*** | ***12 (11.0)*** |
|  |  | No | 2658 (84.1) | *2043 (84.9)* | *124 (81.6)* | *>5 (80.0)* | *376 (81.6)* | *22 (75.9)* | *89 (81.7)* |
|  |  | Reversed | 213 (6.7) | *156 (6.5)* | *8 (5.3)* | *>5 (20.0)* | *37 (8.0)* | *>5 (10.3)* | *8 (7.3)* |
|  | 85th | **Yes** | **359 (11.4)** | ***253 (10.5)*** | ***23 (15.1)*** | ***0 (0.0)*** | ***61 (13.2)*** | ***7 (24.1)*** | ***15 (13.8)*** |
|  |  | No | 2523 (79.8) | *1946 (80.9)* | *115 (75.7)* | *>5 (80.0)* | *354 (76.8)* | *18 (62.1)* | *86 (78.9)* |
|  |  | Reversed | 279 (8.8) | *206 (8.6)* | *14 (9.2)* | *>5 (20.0)* | *46 (10.0)* | *>5 (13.8)* | *8 (7.3)* |
|  | 80th | **Yes** | **417 (13.2)** | ***296 (12.3)*** | ***23 (15.1)*** | ***>5 (40.0)*** | ***69 (15.0)*** | ***7 (24.1)*** | ***20 (18.3)*** |
|  |  | No | 2410 (76.2) | *1858 (77.3)* | *113 (74.3)* | *>5 (60.0)* | *339 (73.5)* | *19 (65.5)* | *78 (71.6)* |
|  |  | Reversed | 334 (10.6) | *251 (10.4)* | *16 (10.5)* | *0 (0.0)* | *53 (11.5)* | *>5 (10.3)* | *11 (10.1)* |
|  | 75th | **Yes** | **471 (14.9)** | ***339 (14.1)*** | ***24 (15.8)*** | ***>5 (40.0)*** | ***79 (17.1)*** | ***8 (27.6)*** | ***19 (17.4)*** |
|  |  | No | 2324 (73.5) | *1787 (74.3)* | *107 (70.4)* | *>5 (40.0)* | *332 (72.0)* | *18 (62.1)* | *78 (71.6)* |
|  |  | Reversed | 366 (11.6) | *279 (11.6)* | *21 (13.8)* | *>5 (20.0)* | *50 (10.8)* | *>5 (10.3)* | *12 (11.0)* |
|  | 70th | **Yes** | **512 (16.2)** | ***373 (15.5)*** | ***27 (17.8)*** | ***>5 (20.0)*** | ***84 (18.2)*** | ***8 (27.6)*** | ***19 (17.4)*** |
|  |  | No | 2236 (70.7) | *1717 (71.4)* | *102 (67.1)* | *>5 (60.0)* | *320 (69.4)* | *19 (65.5)* | *75 (68.8)* |
|  |  | Reversed | 413 (13.1) | *315 (13.1)* | *23 (15.1)* | *>5 (20.0)* | *57 (12.4)* | *>5 (6.9)* | *15 (13.8)* |
|  | 65th | **Yes** | **544 (17.2)** | ***411 (17.1)*** | ***27 (17.8)*** | ***>5 (20.0)*** | ***79 (17.1)*** | ***8 (27.6)*** | ***18 (16.5)*** |
|  |  | No | 2194 (69.4) | *1676 (69.7)* | *103 (67.8)* | *>5 (60.0)* | *318 (69.0)* | *19 (65.5)* | *75 (68.8)* |
|  |  | Reversed | 423 (13.4) | *318 (13.2)* | *22 (14.5)* | *>5 (20.0)* | *64 (13.9)* | *>5 (6.9)* | *16 (14.7)* |
|  | 60th | **Yes** | **565 (17.9)** | ***428 (17.8)*** | ***29 (19.1)*** | ***>5 (20.0)*** | ***81 (17.6)*** | ***8 (27.6)*** | ***18 (16.5)*** |
|  |  | No | 2167 (68.6) | *1652 (68.7)* | *100 (65.8)* | *>5 (60.0)* | *315 (68.3)* | *19 (65.5)* | *78 (71.6)* |
|  |  | Reversed | 429 (13.6) | *325 (13.5)* | *23 (15.1)* | *>5 (20.0)* | *65 (14.1)* | *>5 (6.9)* | *13 (11.9)* |
|  | 55th | **Yes** | **578 (18.3)** | ***441 (18.3)*** | ***27 (17.8)*** | ***>5 (20.0)*** | ***83 (18.0)*** | ***8 (27.6)*** | ***18 (16.5)*** |
|  |  | No | 2149 (68.0) | *1633 (67.9)* | *105 (69.1)* | *>5 (60.0)* | *307 (66.6)* | *19 (65.5)* | *82 (75.2)* |
|  |  | Reversed | 434 (13.7) | *331 (13.8)* | *20 (13.2)* | *>5 (20.0)* | *71 (15.4)* | *>5 (6.9)* | *9 (8.3)* |

# Supplementary Table 4. Between individual and within twin pair associations of the cumulative load of early medical adversities and ASD and autistic symptom cut-offs

| **Between individuals** | **Unadjusted** | | | **Adjusted^1^** | | | | | |
| --- | --- | --- | --- | --- | --- | --- | --- | --- | --- |
| Outcome/ Percentiles |  | Odds Ratio (95% CI) |  | Odds Ratio (95% CI) | | | Exposure level diff significance | | |
|  | One exposure | Two exposures | Three exposures | One exposure | Two exposures | Three exposures | 1 to 2 | 1 to 3 | 2 to 3 |
| ASD diagnosis | 1.20 (0.97—1.50) | 1.88 (1.43—2.48)*** | 3.53 (1.91—6.55)*** | 1.17 (0.94—1.45) | 1.88 (1.42—2.48)*** | 3.33 (1.79—6.20)*** | ** | ** | . |
| 95th | 1.49 (1.31—1.69)*** | 1.76 (1.47—2.12)*** | 3.31 (2.16—5.06)*** | 1.45 (1.28—1.65)*** | 1.68 (1.40—2.02)*** | 3.39 (2.20—5.24)*** |  | *** | ** |
| 90th | 1.37 (1.25—1.51)*** | 1.7 0(1.48—1.95)*** | 2.44 (1.70—3.51)*** | 1.34 (1.22—1.48)*** | 1.64 (1.43—1.88)*** | 2.5 0(1.72—3.62)*** | * | ** | * |
| 85th | 1.25 (1.15—1.36)*** | 1.59 (1.42—1.79)*** | 2.53 (1.85—3.48)*** | 1.23 (1.13—1.33)*** | 1.54 (1.37—1.74)*** | 2.59 (1.87—3.57)*** | *** | *** | ** |
| 80th | 1.21 (1.13—1.31)*** | 1.48 (1.33—1.65)*** | 2.59 (1.93—3.49)*** | 1.19 (1.10—1.28)*** | 1.44 (1.29—1.60)*** | 2.65 (1.95—3.59)*** | ** | *** | *** |
| 75th | 1.20 (1.12—1.28)*** | 1.45 (1.31—1.60)*** | 2.36 (1.77—3.16)*** | 1.17 (1.10—1.26)*** | 1.41 (1.27—1.56)*** | 2.40 (1.78—3.23)*** | ** | *** | *** |
| 70th | 1.20 (1.12—1.28)*** | 1.42 (1.29—1.57)*** | 2.22 (1.67—2.96)*** | 1.18 (1.10—1.26)*** | 1.38 (1.25—1.53)*** | 2.26 (1.68—3.03)*** | ** | *** | ** |
| 65th | 1.19 (1.12—1.26)*** | 1.39 (1.27—1.53)*** | 2.05 (1.53—2.73)*** | 1.17 (1.10—1.24)*** | 1.36 (1.23—1.49)*** | 2.08 (1.55—2.79)*** | ** | *** | ** |
| 60th | 1.19 (1.12—1.27)*** | 1.41 (1.28—1.55)*** | 2.05 (1.53—2.74)*** | 1.17 (1.10—1.25)*** | 1.37 (1.25—1.51)*** | 2.08 (1.55—2.79)*** | ** | *** | ** |
| 55th | 1.22 (1.14—1.29)*** | 1.42 (1.30—1.56)*** | 2.09 (1.55—2.80)*** | 1.2 0(1.13—1.27)*** | 1.39 (1.26—1.53)*** | 2.12 (1.57—2.86)*** | ** | *** | ** |
| **Within twins** |  |  |  |  |  |  |  |  |  |
| ASD diagnosis | 0.90 (0.58—1.38) | 1.04 (0.45—2.39) | 1.95 (0.47—8.17) | 0.92 (0.60—1.42) | 0.91 (0.39—2.16) | 2.39 (0.62—9.24) |  |  |  |
| 95th | 1.51 (1.14—1.98)** | 1.92 (1.09—3.37)* | 6.59 (1.68—25.91)** | 1.52 (1.14—2.03)** | 2.03 (1.16—3.58)* | 7.36 (1.99—27.18)** |  | * | * |
| 90th | 1.34 (1.09—1.64)** | 1.81 (1.22—2.70)** | 2.20 (0.96—5.05) | 1.37 (1.11—1.70)** | 1.91 (1.28—2.87)** | 2.52 (1.07—5.92)* | . |  |  |
| 85th | 1.22 (1.02—1.46)* | 1.58 (1.11—2.24)* | 2.44 (1.16—5.16)* | 1.24 (1.03—1.50)* | 1.63 (1.13—2.33)** | 2.61 (1.22—5.6)* |  | . |  |
| 80th | 1.18 (1.00—1.39)* | 1.53 (1.10—2.12)* | 2.93 (1.48—5.81)** | 1.21 (1.02—1.43)* | 1.55 (1.11—2.17)* | 3.11 (1.53—6.30)** |  | ** | * |
| 75th | 1.18 (1.01—1.38)* | 1.65 (1.20—2.27)** | 2.68 (1.39—5.18)** | 1.20 (1.02—1.40)* | 1.65 (1.19—2.29)** | 2.82 (1.43—5.58)** | * | * |  |
| 70th | 1.16 (1.00—1.34). | 1.58 (1.17—2.14)** | 2.26 (1.19—4.26)* | 1.17 (1.01—1.36)* | 1.57 (1.15—2.14)** | 2.29 (1.18—4.45)* | * | * |  |
| 65th | 1.27 (1.10—1.47)*** | 1.52 (1.13—2.06)** | 2.05 (1.09—3.84)* | 1.28 (1.11—1.49)*** | 1.49 (1.10—2.03)* | 2.05 (1.06—3.96)* |  |  |  |
| 60th | 1.30 (1.13—1.49)*** | 1.55 (1.15—2.08)** | 2.46 (1.27—4.75)** | 1.31 (1.14—1.52)*** | 1.52 (1.13—2.06)** | 2.59 (1.32—5.10)** |  | * |  |
| 55th | 1.32 (1.15—1.52)*** | 1.51 (1.12—2.03)** | 3.22 (1.57—6.59)** | 1.35 (1.17—1.55)*** | 1.50 (1.11—2.02)** | 3.45 (1.66—7.15)*** |  | * | * |

| .=p<0.1, *=p<0.05, **=p<0.01, ***=p< 0.001. *ASD* autism spectrum disorder, *CI* confidence interval. |
| --- |
| ^1^ Adjusted for sex and birthyear for between individual regressions, and adjusted for familial confounding and sex for within twin pair regressions |

# Supplementary Table 5. Split by sex – Associations between the cumulative exposure load of early medical factors and a diagnosis of ASD, and being above each percentile cut-off of autistic symptoms, respectively. N*=* 10,254 twin pairs, excluding opposite sex twins.

| **Between individuals^1^** | **Female** | | | **Male** | | |
| --- | --- | --- | --- | --- | --- | --- |
| Outcome/ Percentiles |  | Odds Ratio (95% CI) |  | Odds Ratio (95% CI) | | |
|  | One exposure | Two exposures | Three exposures | One exposure | Two exposures | Three exposures |
| ASD diagnosis | 0.75 (0.39–1.45) | 1.96 (1.12–3.43)* | 2.90 (0.89–9.47) | 1.23 (0.88–1.71) | 1.54 (1.06–2.23)* | 2.86 (1.31–6.24)** |
| 95th | 1.48 (1.10–1.98)** | 1.68 (1.18–2.38)** | 3.01 (1.37–6.64)** | 1.23 (1.00–1.52) | 1.49 (1.17–1.90)** | 3.52 (2.08–5.94)*** |
| 90th | 1.32 (1.07–1.62)** | 1.62 (1.27–2.07)*** | 2.17 (1.17–4.03)* | 1.14 (0.97–1.33) | 1.38 (1.15–1.67)*** | 2.45 (1.54–3.9)*** |
| 85th | 1.26 (1.06–1.50)** | 1.49 (1.21–1.84)*** | 1.96 (1.14–3.34)* | 1.03 (0.90–1.19) | 1.31 (1.11–1.54)** | 2.82 (1.88–4.25)*** |
| 80th | 1.17 (1.01–1.37)* | 1.44 (1.20–1.74)*** | 2.06 (1.28–3.32)** | 1.08 (0.96–1.23) | 1.22 (1.05–1.41)* | 2.40 (1.61–3.58)*** |
| 75th | 1.18 (1.03–1.36)* | 1.38 (1.16–1.64)*** | 1.66 (1.04–2.63)* | 1.11 (0.99–1.25) | 1.16 (1.01–1.34)* | 2.13 (1.44–3.15)*** |
| 70th | 1.22 (1.07–1.39)** | 1.39 (1.19–1.64)*** | 1.87 (1.22–2.88)** | 1.08 (0.97–1.21) | 1.13 (0.98–1.30) | 1.90 (1.28–2.80)** |
| 65th | 1.20 (1.06–1.35)** | 1.31 (1.12–1.53)*** | 1.58 (1.03–2.42)* | 1.11 (0.99–1.23) | 1.15 (1.01–1.32)* | 1.91 (1.29–2.83)** |
| 60th | 1.16 (1.03–1.31)* | 1.31 (1.13–1.53)*** | 1.68 (1.11–2.55)* | 1.12 (1.01–1.25)* | 1.18 (1.03–1.35)* | 1.89 (1.28–2.81)** |
| 55th | 1.20 (1.06–1.35)** | 1.29 (1.11–1.49)*** | 1.93 (1.27–2.92)** | 1.16 (1.04–1.29)** | 1.19 (1.05–1.37)** | 1.77 (1.19–2.65)** |
| **Within twins^1^** |  |  |  |  |  |  |
| ASD diagnosis | 1.24 (0.34–4.56) | 1.09 (0.29–4.11) | NA | 1.53 (0.71–3.29) | 0.69 (0.26–1.84) | 3.20 (0.34–30.45) |
| 95th | 1.64 (0.86–3.12) | 2.20 (0.95–5.12) | 3.12 (0.59–16.54) | 1.39 (0.84–2.30) | 2.13 (1.07–4.26)* | 4.92 (1.06–22.71)* |
| 90th | 1.30 (0.82–2.06) | 1.56 (0.90–2.71) | 1.74 (0.55–5.52) | 1.19 (0.82–1.71) | 1.64 (1.01–2.64)* | 1.87 (0.65–5.35) |
| 85th | 1.41 (0.94–2.11) | 1.27 (0.79–2.05) | 2.17 (0.73–6.44) | 1.03 (0.75–1.43) | 1.33 (0.87–2.03) | 3.19 (1.06–9.57)* |
| 80th | 1.18 (0.83–1.66) | 1.26 (0.82–1.93) | 2.52 (0.88–7.22) | 1.07 (0.80–1.44) | 1.34 (0.90–1.99) | 2.35 (0.88–6.31) |
| 75th | 1.28 (0.92–1.77) | 1.27 (0.84–1.93) | 1.97 (0.72–5.36) | 1.07 (0.82–1.41) | 1.45 (0.98–2.13) | 2.37 (0.97–5.78) |
| 70th | 1.33 (0.98–1.82) | 1.36 (0.92–2.03) | 2.76 (0.96–7.91) | 1.06 (0.82–1.37) | 1.34 (0.91–1.97) | 1.83 (0.79–4.23) |
| 65th | 1.26 (0.94–1.69) | 1.20 (0.82–1.75) | 1.16 (0.43–3.14) | 1.23 (0.95–1.59) | 1.42 (0.97–2.07) | 2.71 (1.14–6.44)* |
| 60th | 1.18 (0.89–1.57) | 1.13 (0.77–1.63) | 1.66 (0.67–4.14) | 1.34 (1.03–1.75)* | 1.66 (1.14–2.40)** | 3.98 (1.55–10.20)** |
| 55th | 1.23 (0.93–1.62) | 1.14 (0.78–1.66) | 1.84 (0.74–4.57) | 1.26 (0.97–1.64) | 1.36 (0.95–1.95) | 2.59 (0.98–6.87) |

*=p<0.05, **=p<0.01, ***=p< 0.001

^1^ Adjusted for birthyear for between individual regressions and adjusted for familial confounding for within twin pair regressions.

# Supplementary Table 6. Birthyear adjusted between individual and familial confounding adjusted within twin-pair associations between the cumulative exposure load of early medical factors and a diagnosis of ASD, and being above each percentile cut-off of autistic symptoms, with each factor removed respectively.

| **Between individuals** | **Malformation & Hypoxia** | | **LBW & Hypoxia** | | **LBW & Malformation** | |
| --- | --- | --- | --- | --- | --- | --- |
| Outcome/ Percentiles | Odds Ratio (95% CI) | | Odds Ratio (95% CI) | | Odds Ratio (95% CI) | |
|  | One exposure | Two exposures | One exposure | Two exposures | One exposure | Two exposures |
| ASD diagnosis | 1.4 (1.15–1.70)*** | 2.46 (1.45–4.19)*** | 1.11 (0.88–1.40) | 2.14 (1.64–2.80)*** | 1.58 (1.26–1.96)*** | 2.61 (1.45–4.70)** |
| 95th | 1.47 (1.3–1.66)*** | 2.37 (1.65–3.40)*** | 1.38 (1.21–1.58)*** | 1.77 (1.48–2.13)*** | 1.53 (1.33–1.76)*** | 3.49 (2.42–5.04)*** |
| 90th | 1.39 (1.27–1.52)*** | 1.99 (1.49–2.67)*** | 1.27 (1.15–1.41)*** | 1.65 (1.43–1.89)*** | 1.46 (1.32–1.62)*** | 2.65 (1.94–3.63)*** |
| 85th | 1.30 (1.2–1.40)*** | 1.75 (1.34–2.27)*** | 1.17 (1.08–1.28)*** | 1.64 (1.45–1.84)*** | 1.39 (1.27–1.52)*** | 2.65 (2.01–3.49)*** |
| 80th | 1.24 (1.16–1.33)*** | 1.82 (1.43–2.32)*** | 1.15 (1.06–1.24)*** | 1.54 (1.38–1.72)*** | 1.33 (1.22–1.44)*** | 2.53 (1.95–3.29)*** |
| 75th | 1.24 (1.16–1.33)*** | 1.77 (1.40–2.22)*** | 1.17 (1.09–1.26)*** | 1.51 (1.36–1.68)*** | 1.27 (1.17–1.37)*** | 2.16 (1.67–2.79)*** |
| 70th | 1.24 (1.17–1.32)*** | 1.77 (1.41–2.22)*** | 1.17 (1.09–1.25)*** | 1.46 (1.32–1.62)*** | 1.24 (1.15–1.33)*** | 2.06 (1.60–2.64)*** |
| 65th | 1.23 (1.16–1.31)*** | 1.71 (1.37–2.14)*** | 1.15 (1.08–1.23)*** | 1.41 (1.28–1.56)*** | 1.21 (1.13–1.30)*** | 1.90 (1.48–2.44)*** |
| 60th | 1.23 (1.16–1.31)*** | 1.76 (1.41–2.20)*** | 1.15 (1.08–1.23)*** | 1.43 (1.30–1.57)*** | 1.24 (1.15–1.33)*** | 1.88 (1.46–2.41)*** |
| 55th | 1.27 (1.20–1.34)*** | 1.67 (1.34–2.08)*** | 1.18 (1.11–1.26)*** | 1.44 (1.31–1.59)*** | 1.23 (1.15–1.32)*** | 1.91 (1.48–2.46)*** |
| **Within twins** |  |  |  |  |  |  |
| ASD diagnosis | 0.91 (0.59–1.41) | 1.83 (0.6–5.61) | 0.86 (0.52–1.4) | 0.99 (0.38–2.62) | 1.00 (0.60–1.66) | 2.15 (0.64–7.30) |
| 95th | 1.52 (1.13–2.05)** | 3.22 (1.14–9.11)* | 1.19 (0.87–1.64) | 1.71 (0.92–3.19) | 1.61 (1.12–2.32)** | 3.96 (1.53–10.26)** |
| 90th | 1.32 (1.07–1.64)** | 2.03 (1.04–3.97)* | 1.16 (0.92–1.46) | 1.52 (0.96–2.40) | 1.59 (1.23–2.05)*** | 2.53 (1.29–4.95)** |
| 85th | 1.18 (0.98–1.43) | 1.69 (0.93–3.06) | 1.12 (0.91–1.38) | 1.40 (0.94–2.09) | 1.43 (1.14–1.80)** | 3.43 (1.79–6.58)*** |
| 80th | 1.15 (0.97–1.37) | 2.15 (1.24–3.75)** | 1.12 (0.93–1.35) | 1.44 (0.99–2.09) | 1.37 (1.11–1.68)** | 3.31 (1.78–6.16)*** |
| 75th | 1.19 (1.01–1.40)* | 1.98 (1.18–3.35)* | 1.20 (1.01–1.43)* | 1.67 (1.16–2.41)** | 1.24 (1.02–1.51)* | 2.43 (1.35–4.36)** |
| 70th | 1.21 (1.04–1.40)* | 1.94 (1.19–3.18)** | 1.16 (0.99–1.37) | 1.48 (1.04–2.12)* | 1.15 (0.95–1.38) | 2.07 (1.16–3.70)* |
| 65th | 1.31 (1.12–1.52)*** | 1.72 (1.06–2.81)* | 1.25 (1.06–1.47)** | 1.44 (1.01–2.04)* | 1.17 (0.97–1.40) | 1.95 (1.10–3.48)* |
| 60th | 1.33 (1.15–1.54)*** | 1.96 (1.19–3.20)** | 1.25 (1.06–1.46)** | 1.40 (0.99–1.96) | 1.23 (1.03–1.48)* | 2.64 (1.47–4.75)** |
| 55th | 1.33 (1.15–1.54)*** | 2.13 (1.29–3.51)** | 1.28 (1.10–1.50)** | 1.48 (1.05–2.08)* | 1.23 (1.03–1.47)* | 2.71 (1.48–4.97)** |
| *=p<0.05, **=p<0.01, ***=p< 0.001 | | | | | | |
| *LBW* gestational age adjusted low birth weight, *ASD* autism spectrum disorder, *CI* confidence interval | | | | | | |

# Supplementary Figure 1. Cumulative exposure with each factor removed respectively

Birthyear adjusted between individual (upper panels) and familial confounding adjusted within twin-pair (lower panels) associations between the cumulative exposure load of early medical factors and a diagnosis of ASD and being above each percentile cut-off of autistic symptoms. Forest plots illustrating odds ratios (ORs, dots) and 95% confidence interval (CI, bars) from conditional regressions with each factor removed respectively (panels from left to right). *LBW* low birth weight.

# Supplementary Table 7. Birthyear adjusted between individual and familial confounding adjusted within twin-pair associations for each exposure respectively and a diagnosis of ASD, and being above each percentile cut-off of autistic symptoms

| **Between individuals** | **LBW** | **Malformations** | **Hypoxia** |
| --- | --- | --- | --- |
| Outcome/ Percentiles | Odds Ratio (95% CI) | Odds Ratio (95% CI) | Odds Ratio (95% CI) |
|  |  |  |
| ASD diagnosis | 1.66 (1.32—2.09)*** | 1.59 (1.12—2.26)** | 1.45 (1.19—1.76)*** |
| 95th | 1.63 (1.41—1.89)*** | 1.89 (1.52—2.35)*** | 1.45 (1.28—1.64)*** |
| 90th | 1.5 (1.34—1.68)*** | 1.72 (1.46—2.04)*** | 1.38 (1.25—1.51)*** |
| 85th | 1.48 (1.35—1.63)*** | 1.53 (1.32—1.77)*** | 1.29 (1.19—1.39)*** |
| 80th | 1.4 (1.29—1.53)*** | 1.48 (1.29—1.69)*** | 1.24 (1.16—1.34)*** |
| 75th | 1.34 (1.23—1.46)*** | 1.31 (1.15—1.49)*** | 1.27 (1.19—1.36)*** |
| 70th | 1.3 (1.2—1.4)*** | 1.31 (1.16—1.49)*** | 1.27 (1.19—1.35)*** |
| 65th | 1.26 (1.16—1.36)*** | 1.3 (1.15—1.47)*** | 1.25 (1.18—1.34)*** |
| 60th | 1.27 (1.18—1.37)*** | 1.34 (1.19—1.52)*** | 1.25 (1.18—1.33)*** |
| 55th | 1.27 (1.18—1.37)*** | 1.33 (1.18—1.5)*** | 1.29 (1.21—1.37)*** |
| **Within twins** |  |  |  |
| ASD diagnosis | 0.98 (0.5—1.94) | 1.26 (0.67—2.35) | 0.94 (0.58—1.53) |
| 95th | 1.34 (0.88—2.03) | 2.51 (1.51—4.17)*** | 1.27 (0.92—1.75) |
| 90th | 1.32 (0.97—1.81) | 1.94 (1.38—2.73)*** | 1.17 (0.92—1.48) |
| 85th | 1.43 (1.08—1.89)* | 1.68 (1.24—2.29)*** | 1.05 (0.86—1.3) |
| 80th | 1.37 (1.05—1.77)* | 1.6 (1.21—2.12)*** | 1.08 (0.89—1.31) |
| 75th | 1.32 (1.03—1.69)* | 1.32 (1.01—1.73)* | 1.22 (1.02—1.45)* |
| 70th | 1.14 (0.9—1.44) | 1.34 (1.04—1.73)* | 1.22 (1.03—1.44)* |
| 65th | 1.11 (0.88—1.4) | 1.38 (1.07—1.77)* | 1.28 (1.09—1.51)** |
| 60th | 1.13 (0.9—1.43) | 1.59 (1.24—2.04)*** | 1.25 (1.07—1.47)** |
| 55th | 1.14 (0.91—1.44) | 1.54 (1.21—1.97)*** | 1.3 (1.11—1.52)** |
| *=p<0.05, **=p<0.01, ***=p< 0.001 | |  |  |
| *LBW* gestational age adjusted low birth weight | |  |  |

# Supplementary Figure 2. Odds Ratios for each exposure respectively

Birthyear adjusted between individual (left panel) and familial confounding adjusted within twin-pair (right panel) associations between each exposure and a diagnosis of ASD and being above each percentile cut-off of autistic symptoms. Forest plots illustrating odds ratios (ORs, dots) and 95% confidence interval (CI, bars) from conditional regressions. *LBW* low birth weight.

# Supplementary Table 8. Between individual and within twin pair associations between the cumulative load of early medical factors and ASD and autistic symptom cut-offs. *n=93* twin pairs excluded, were either one or both twins in the pair had received a diagnosis of twin-to-twin transfusion syndrome.

| **Between individuals** | **Unadjusted** | | | **Adjusted^1^** | | |
| --- | --- | --- | --- | --- | --- | --- |
| Outcome/ Percentiles |  | Odds Ratio (95% CI) |  | Odds Ratio (95% CI) | | |
|  | One exposure | Two exposures | Three exposures | One exposure | Two exposures | Three exposures |
| ASD diagnosis | 1.21 (0.97–1.50) | 1.93 (1.46–2.54)*** | 3.62 (1.95–6.71)*** | 1.17 (0.94–1.46) | 1.91 (1.45–2.53)*** | 3.40 (1.82–6.33)*** |
| 95th | 1.45 (1.28–1.65)*** | 1.77 (1.47–2.12)*** | 3.37 (2.20–5.16)*** | 1.42 (1.25–1.62)*** | 1.69 (1.41–2.04)*** | 3.45 (2.23–5.33)*** |
| 90th | 1.34 (1.22–1.47)*** | 1.69 (1.47–1.94)*** | 2.49 (1.73–3.58)*** | 1.31 (1.19–1.45)*** | 1.63 (1.42–1.87)*** | 2.54 (1.75–3.70)*** |
| 85th | 1.24 (1.14–1.34)*** | 1.59 (1.41–1.79)*** | 2.60 (1.90–3.58)*** | 1.21 (1.12–1.32)*** | 1.54 (1.37–1.74)*** | 2.65 (1.92–3.67)*** |
| 80th | 1.20 (1.12–1.30)*** | 1.48 (1.32–1.65)*** | 2.68 (1.99–3.61)*** | 1.18 (1.10–1.27)*** | 1.44 (1.28–1.60)*** | 2.73 (2.00–3.71)*** |
| 75th | 1.20 (1.12–1.28)*** | 1.45 (1.31–1.61)*** | 2.45 (1.83–3.28)*** | 1.17 (1.10–1.26)*** | 1.41 (1.27–1.57)*** | 2.48 (1.84–3.35)*** |
| 70th | 1.19 (1.12–1.27)*** | 1.42 (1.29–1.57)*** | 2.31 (1.73–3.09)*** | 1.17 (1.10–1.25)*** | 1.39 (1.25–1.53)*** | 2.35 (1.74–3.16)*** |
| 65th | 1.18 (1.11–1.26)*** | 1.39 (1.26–1.53)*** | 2.13 (1.60–2.85)*** | 1.16 (1.09–1.24)*** | 1.35 (1.22–1.49)*** | 2.16 (1.61–2.91)*** |
| 60th | 1.19 (1.12–1.26)*** | 1.41 (1.28–1.55)*** | 2.15 (1.60–2.88)*** | 1.17 (1.10–1.24)*** | 1.37 (1.25–1.51)*** | 2.18 (1.62–2.94)*** |
| 55th | 1.21 (1.14–1.28)*** | 1.42 (1.29–1.56)*** | 2.11 (1.56–2.84)*** | 1.19 (1.12–1.27)*** | 1.39 (1.26–1.53)*** | 2.14 (1.58–2.89)*** |
| **Within twins** |  |  |  |  |  |  |
| ASD diagnosis | 0.90 (0.58–1.38) | 1.04 (0.45–2.39) | 1.95 (0.47–8.17) | 0.92 (0.60–1.42) | 0.91 (0.39–2.16) | 2.39 (0.62–9.24) |
| 95th | 1.44 (1.09–1.90)** | 1.87 (1.06–3.31)* | 6.40 (1.62–25.24)** | 1.45 (1.08–1.93)* | 1.98 (1.12–3.51)* | 7.12 (1.92–26.40)** |
| 90th | 1.29 (1.06–1.59)* | 1.74 (1.17–2.60)** | 2.30 (0.98–5.39) | 1.33 (1.08–1.65)** | 1.84 (1.22–2.76)** | 2.67 (1.11–6.40)* |
| 85th | 1.21 (1.01–1.45)* | 1.53 (1.07–2.18)* | 2.57 (1.20–5.53)* | 1.22 (1.01–1.48)* | 1.57 (1.09–2.26)* | 2.76 (1.26–6.04)* |
| 80th | 1.17 (0.99–1.38) | 1.48 (1.06–2.06)* | 3.32 (1.62–6.82)** | 1.19 (1.01–1.42)* | 1.50 (1.07–2.10)* | 3.56 (1.70–7.45)*** |
| 75th | 1.18 (1.01–1.38)* | 1.58 (1.14–2.18)** | 2.95 (1.49–5.86)** | 1.19 (1.02–1.40)* | 1.58 (1.14–2.19)** | 3.14 (1.54–6.39)** |
| 70th | 1.15 (1.00–1.33) | 1.54 (1.14–2.10)** | 2.48 (1.29–4.80)** | 1.16 (1.00–1.35) | 1.52 (1.12–2.08)** | 2.54 (1.28–5.03)** |
| 65th | 1.26 (1.09–1.45)** | 1.51 (1.11–2.04)** | 2.26 (1.18–4.32)* | 1.27 (1.10–1.47)** | 1.47 (1.08–2.01)* | 2.28 (1.16–4.48)* |
| 60th | 1.29 (1.12–1.48)*** | 1.52 (1.13–2.05)** | 2.76 (1.39–5.48)** | 1.31 (1.13–1.51)*** | 1.49 (1.10–2.02)** | 2.93 (1.45–5.93)** |
| 55th | 1.31 (1.14–1.50)*** | 1.51 (1.12–2.03)** | 3.37 (1.60–7.07)** | 1.33 (1.15–1.54)*** | 1.50 (1.11–2.02)** | 3.62 (1.71–7.70)*** |

*=p<0.05, **=p<0.01, ***=p< 0.001. *ASD* autism spectrum disorder, *CI* confidence interval.

^1^ Adjusted for sex and birthyear for between individual regressions, and adjusted for familial confounding and sex for within twin pair regressions

# Supplementary Figure 3. Cumulative exposure excluding twins with twin-to-twin transfusion syndrome

Between individual (upper panels) and within twin-pair (lower panels) associations between the cumulative exposure load of early medical factors and a diagnosis of ASD, and being above each percentile cut-off of autistic symptoms, respectively. Forest plots illustrating odds ratios (ORs, dots) and 95% confidence interval (CI, bars) for unadjusted associations to each exposure level (left panels), and sex and birthyear adjusted between individual (upper right panel) and familial confounding and sex adjusted (lower right panel) within twin associations.
